# Lymphocyte Medium-Chain Acyl-CoA Dehydrogenase Activity and Its Potential as a Diagnostic Confirmation Tool in Newborn Screening Cases

**DOI:** 10.3390/jcm11102933

**Published:** 2022-05-23

**Authors:** Patricia Alcaide, Isaac Ferrer-López, Leticia Gutierrez, Fatima Leal, Elena Martín-Hernández, Pilar Quijada-Fraile, Marcello Bellusci, Ana Moráis, Consuelo Pedrón-Giner, Dolores Rausell, Patricia Correcher, María Unceta, Sinziana Stanescu, Magdalena Ugarte, Pedro Ruiz-Sala, Belén Pérez

**Affiliations:** 1 Centro de Diagnóstico de Enfermedades Moleculares (CEDEM), Universidad Autónoma Madrid, CIBERER, IDIPAZ, 28049 Madrid, Spain; isaac.ferrer@inv.uam.es (I.F.-L.); leticia.gutierrez@inv.uam.es (L.G.); fleal@cbm.csic.es (F.L.); mugarte@cbm.csic.es (M.U.); pedro.ruiz@inv.uam.es (P.R.-S.); bperez@cbm.csic.es (B.P.); 2 Centro de Referencia Nacional (CSUR) y Europeo (MetabERN) para Enfermedades Metabólicas, Hospital Universitario 12 de Octubre, 28041 Madrid, Spain; emartinhernandez@salud.madrid.org (E.M.-H.); pilar.quijadaf@salud.madrid.org (P.Q.-F.); marcello.bellusci@salud.madrid.org (M.B.); 3 Unidad de Nutrición Infantil y Enfermedades Metabólicas, Hospital Universitario Infantil La Paz, 28046 Madrid, Spain; morais.lopez@salud.madrid.org; 4 Sección de Gastroenterología y Nutrición, Hospital Infantil Universitario Niño Jesús, 28009 Madrid, Spain; consuelocarmen.pedron@salud.madrid.org; 5 Laboratorio de Metabolopatías, Servicio de Análisis Clínicos, Hospital Universitario La Fe, 46026 Valencia, Spain; rausell_dol@gva.es (D.R.); correcher_pat@gva.es (P.C.); 6 Análisis Clínicos, Servicio de Bioquímica, Unidad de Enfermedades Metabólicas, Hospital Universitario de Cruces, 48903 Barakaldo, Spain; maria.uncetasuarez@osakidetza.eus; 7 Servicio de Pediatría, Unidad de Enfermedades Metabólicas, Hospital Universitario Ramón y Cajal, IRYCIS, 28034 Madrid, Spain; sinziana.stanescu@salud.madrid.org

**Keywords:** acylcarnitines, lymphocyte enzyme activity, medium-chain acyl-CoA dehydrogenase, newborn screening, fatty acid oxidation

## Abstract

The determination of acylcarnitines (AC) in dried blood spots (DBS) by tandem mass spectrometry in newborn screening (NBS) programs has enabled medium-chain acyl-coA dehydrogenase deficiency (MCADD) to be identified in presymptomatic newborns. Nevertheless, different confirmatory tests must be performed to confirm the diagnosis. In this work, we have collected and analyzed the NBS results and confirmatory test results (plasma AC, molecular findings, and lymphocyte MCAD activity) of forty individuals, correlating them with clinical outcomes and treatment, with the aim of obtaining useful diagnostic information that could be applied in the follow-up of the patients. Our results led us to classify patients into two groups. The first group (14 cases) had high increased octanoylcarnitine (C8) levels, biallelic pathogenic variants, and severe impaired enzyme activity (<10% of the intra-assay control (IAC)); all of these cases received nutritional therapy and required carnitine supplementation during follow-up, representing the most severe form of the disease. The second group (16 patients) was a heterogeneous group presenting moderate increases in C8, biallelic likely pathogenic/pathogenic variants, and intermediate activity (<41% IAC). All of them are currently asymptomatic and could be considered as having a milder form of the disease. Finally, eight cases presented a normal–mild increase in plasma C8, with only one pathogenic variant detected, and high–intermediate residual activity (15–100%). Based on our results, we confirm that combined evaluation of acylcarnitine profiles, genetic findings, and residual enzyme activities proves useful in predicting the risk of future metabolic decompensation, in making decisions regarding future treatment or follow-up, and also in confirming the clinical effects of unknown clinical variants.

## 1. Introduction

Medium-chain acyl-CoA dehydrogenase (MCAD, EC1.3.8.7) is a mitochondrial matrix flavoprotein essential for the beta-oxidation of medium-chain fatty acids, and its deficiency is the most commonly known genetic disorder of fatty acid oxidation (FAO) (OMIM 201450). In Iberia, and influenced by Portuguese population data, MCADD has a birth prevalence of 1:11,945, which is one of the highest reported in European countries, alongside the U.K., Denmark, and the Netherlands [1].

MCAD deficiency (MCADD) is associated with hypoketotic hypoglycemia, lethargy, coma seizures, or hepatic symptoms that are predominantly related to intercurrent illnesses or prolonged fasting [2]. Sudden death related to heartbeat disorders may also occur in adults [3] or neonates [4]. The disease usually manifests in the first years of life [5], although first presentation in adulthood has also been described [6]. There is a risk of neurological impairment after an acute metabolic decompensation [5,7]. Even though MCADD is one of the most common and better-known inborn errors of metabolism, there are still significant gaps in the understanding of the natural history of this condition, including its effects on the growth and development of patients [8].

The determination of acylcarnitines (AC) in dried blood spots (DBS) by tandem mass spectrometry (MS/MS) in newborn screening (NBS) programs has enabled MCADD to be identified in presymptomatic newborns through the elevation of its primary marker C8-acylcarnitine, with lesser increases in C6- and C10-acylcarnitines. The cutoff values for C8 differ between NBS programs and may be combined with elevated secondary markers including C0, C2, C10:1, and the C8/C2 and C8/C10 ratios in presumptive positive cases to improve NBS sensitivity and positive predictive value [9]. Finally, the patients being considered as probable positives require follow-up, biochemical testing, and, finally, genetic analysis to confirm MCADD. Confirmatory testing includes plasma AC, urine organic acid, and acylglycine analysis (hexanoylglycine and suberylglycine). If the test results support the likelihood of MCADD, genetic studies are mandatory to establish a diagnosis [10].

In many cases, a variant of unknown significance (VUS) or just one single exonic pathogenic variant is identified. In such cases, the MCAD enzymatic activity must be used to aid in disease confirmation and also to provide clues to predict possible clinical outcomes, as occurs in other fatty acid oxidation (FAO) defects [11]. In addition, clinical follow-up parameters are difficult to interpret because early diagnosis and treatment influence the natural clinical course of subjects with variant *ACADM* genotypes. It could be hypothesized that residual MCAD enzyme activity is a prognostic parameter in the risk stratification of patients with variant *ACADM* genotypes. It can be questioned, however, whether subjects with variant *ACADM* genotypes have the same clinical risks compared to patients with classical *ACADM* genotypes, or even whether they should be regarded as patients at all [12].

There is evidence that residual enzyme function correlates with the expected phenotype. Different biallelic and monoallelic variants in the *ACADM* gene are associated with variable reductions in cellular enzyme function and subsequent differences in the risk of metabolic decompensation [13,14,15]. However, it remains often difficult to evaluate at which level of residual enzyme function there is a risk of a metabolic derangement. Despite the diagnosis, the clinical relevance of mild MCADD remains uncertain [16].

Our laboratory developed a rapid MCAD-VLCAD activity quantification method in lymphocytes, based on LC-MS/MS quantification of octenoyl-CoA (C8:1-CoA) and hydroxyoctanoyl-CoA (C8:OH-CoA), published by ter Veld and Tajima [10,17]. In this work, we present the results of biochemical, enzymatic, and mutational analyses of selected cases detected in the NBS program with MCADD suspicion. Through this work, we contribute to determining the severity of some pathogenic variants and confirming the carrier status of cases with only one variant detected by exome sequencing.

## 2. Materials and Methods

### 2.1. Patients

A retrospective compilation and analysis was performed on the results of clinical evaluation and confirmatory biochemical/genetic tests carried out on 36 neonates with suspected MCADD that were detected by LC/MS/MS in NBS from 4 autonomous communities in Spain (Madrid, Valencia, Castilla-La Mancha, and Pais Vasco); all of them presented an elevation of C8-acylcarnitine. We also included two patients diagnosed following the onset of symptoms, as expanded newborn screening was not available on their birth date (P8 and P38), one (P17) who was diagnosed by an altered neonatal screening in a sibling, and two parents of NBS patients as obligate carriers (P39 and P40) (Table 1).

Each NBS center had previously independently determined its internal NBS 99.5 percentile cut-off levels and the criteria for positive screening of newborns at 48–72 h of life. If the presence of a target disease is suspected, the screening laboratory immediately informs the physician to request a second sample to check the finding. The identified cases were remitted to their clinical reference units for confirmation of MCADD between 7 and 20 days post-partum, and new samples were then sent to confirmation laboratories. In all cases, the confirmatory tests involved measuring the plasma AC levels, determining the organic acids and acylglycines in urine, and performing a thorough molecular analysis of the *ACADM* gene. In some cases, namely, those with two mutations or those with only one variant of unknown clinical significance, or cases where clinical symptoms did not support the genetic findings, MCAD enzyme activity in lymphocytes was assessed by LC-MS/MS.

### 2.2. Biochemical Analysis

AC levels were measured in plasma obtained between days 8 and 23 except in cases P8 and P38, where it was obtained at 16 months and 10 years of age, respectively, and analyzed as described previously [18]. Organic acids were determined by GC/MS as trimethylsilyl derivatives after urease treatment, and ethyl acetate liquid–liquid extraction without oxymation.

### 2.3. MCAD Enzyme Activity in Lymphocytes

The method used to assess MCAD activity is based on the oxidation of octanoyl-CoA (C8:0-CoA) in the presence of an electron acceptor, ferrocenium hexafluorophosphate (FcPF6), essentially as described in [17]. Lymphocytes were isolated from whole blood (3–5 mL) using Histopaque (Sigma-Aldrich, Deisenhofen, Germany), and the resulting pellet was frozen before use. The lymphocytes were resuspended in a buffer containing 125 mmol/L KH_2_PO_4_, 1 mmol/L EDTA, and 9% Triton X-100, and they were then sonicated twice for 5 min, kept on ice for 30 min and, finally, centrifuged at 12,000 rpm for 10 min. The reaction mixtures contained 5 mmol/L C8:0-CoA and 4 mmol/L FcFP6 in a final volume of 100 mL.

The analysis of C8:0-CoA, 2-octenoyl-CoA (C8:1-CoA) and 3-hydroxyoctanoyl-CoA (C8OH-CoA) reaction products was performed via LC/MS/MS (1290 Agilent Series HPLC, Santa Clara, CA, USA), with the apparatus coupled to an Applied Biosystems 4500 QTrap (Carlsbad, CA, USA). A 5 µL aliquot of each sample was injected onto a Symmetry C18 column (100 mm × 2.1 mm, particle size 3.5 µm, Waters). Ammonium acetate (10 mM) in water (eluent A) and ammonium acetate (10 mM) in methanol (eluent B) were used to make the gradient of the mobile phase as follows: 15% B for 1 min, linearly changed to 45% B over 14 min, then to 100% B over 1 min and held for 4 min. This was subsequently changed from 100 to 15% over 1 min and held for 5 min to re-equilibrate the column. The flow rate was 200 µL/min and the run time was 36 min. Acyl-CoAs were analyzed in positive multiple reaction monitoring (MRM) mode, with MRM transitions of 894/387 for C8:0-CoA, 892/385 for C8:1-CoA and 910/403 for C8OH-CoA.

Quantification was based on the peak area (sum of product areas/(sum of substrate + product areas) ratio) with the initial amount of C8:0-CoA substrate set to 20 nmol [10]. Proteins were quantified by the Lowry method before Triton X-100 was added. Product formation was shown to be linear for up to 15 min and proportional to the amount of protein added in the range of 20–200 mg, with a correlation coefficient of r^2^ = 0.99. The limit of C8:1-CoA or C8OH-CoA detection (signal/noise ratio of >3) was estimated to be 0.015 nmol/min/mg protein. The intra-day reproducibility of MCAD activity was calculated in one control sample, realizing multiple replicates that revealed an imprecision coefficient of 3%, indicative of satisfactory reproducibility in the analysis. VLCAD activity was also measured as previously described in [19], as a control for the correct metabolic conditions of the cells.

### 2.4. Genetic Analysis

Genetic analysis was performed by exome and flanking intronic sequencing of the *ACADM* gene via Sanger sequencing or massive parallel sequencing using either a targeted, customized panel that captures the exome of 119 genes involved in metabolic disorders (Nextera Nature Capture (Illumina, San Diego, CA, USA)), or an extended panel (Clinical-Exome Sequencing TruSight™ One Gene Panel (Illumina)) that includes the exomic region of all the known disease-associated genes described in the OMIM database (2013) (Mendeliome panel). Variants were classified following the ACMG rules [20] using the RefSeq number NM_000016.6.

### 2.5. Statistical Analysis

All statistics performed were determined using t-test (assuming equal variance) with a two-sided alpha value of <0.05.

## 3. Results

### 3.1. Newborn Screening

Fifteen cases presented a significant increase in C8 (1.6–20 µmol/L) in DBS (P1–P7, P9–P12, P25, P26, P27, P28 and P30), and the remaining twenty individuals showed a moderate increase in C8 ranging from (0.17–1.5 µmol/L) (Table 1).

### 3.2. Confirmatory Testing

#### 3.2.1. Biochemical Confirmatory Analysis

Out of 35 cases remitted from NBS programs, or the 3 with MCADD suspicion, 16 cases (P1–P12, P25–P27, and P30) presented, in confirmatory analysis, high increased C8 plasma levels (1–10.8 µmol/L; NV < 0.25), 17 cases (P13–P21, P23–P24, P28, P32–P35, and P38) presented mild increased C8 levels (0.23–1 µmol/L), and five cases (P22, P29, P31, P36, and P37) presented C8 levels with a mean in the normal range. The results of confirmatory AC testing are presented as measurements of means and their range (Table 1).

Acylglycine (hexanoylglycine and suberylglycine) excretion was increased in P1–P12, P26, P27, and P30, and was normal or slightly increased in the remaining cases with two pathogenic variants. Patients with only one variant presented normal excretion, except for P33, who presented a mild increase in some determinations (data not shown).

#### 3.2.2. Genetic Studies

Genetic analysis of the *ACADM* gene was performed on 40 individuals (33 unrelated families), of whom 30 had two biallelic variants (P1–P30), while 8 presented only one variant in the exonic region of the *ACADM* gene (P31–P38). Thirteen different variants and nine different genotypes were found in our cohort.

The most common pathogenic variant described in *ACADM*, c.985A > G, is present in total in 47 unrelated alleles of our cohort of patients (59%), with 12 homozygous patients and 15 compound heterozygous patients. Eight cases were compound heterozygous of c.985A > G and [p.Thr228Asn] (c.683C > A), reported as a conflicting variant in ClinVar, and three patients were detected in combination with [p.Glu43Lys](c.127G > A). Four patients combined c.985A > G with other nucleotide variants: [p.Pro209Leu](626C > T), [p.Cys116Gly] (c.346T > G), [p.Leu84Phe] (c.250C > T), or [p.?] (c.599 + 3A > G) (Table 1). Only three cases did not bear c.985 A > G and were carriers of two different variants (P28–P30). Out of the eight cases with just one exonic variant found, six carried the c.985A > G severe loss-of-function mutation, one presented a severe out-of-frame deletion ([c.449_452delCTGA] p.Thr150Argfs*4, which causes a premature stop codon [21]), and one case presented a missense variation ([p.Gly85Arg] c.253G > C). Patients P39 and P40 are obligate carriers of the c.985A > G mutation (parental progenitors of two confirmed MCAD cases) (Table 1).

All variants but one (c.626C > T) were previously described [16,22], although the pathogenity of some of them was not previously proven by functional studies. The nucleotide change c.626C > T is likely a missense mutation (p.Pro209Leu) and was classified as a VUS in ClinVar according to the AGMD rules and by the Varsome platform [20] as well as c.346T > G.

Regarding our biochemical confirmatory analysis, statistically significant differences in the C8 levels were found via both DBS and plasma confirmatory testing between cases with two pathological variants and patients with only one variant found (mean) (*p* < 0.001).

There were also significant differences in the C8 concentration between homozygotes for the c.985A > G variation and compound heterozygotes with another variation.

#### 3.2.3. Lymphocyte Enzyme Activity

The MCAD activity was measured in lymphocytes from 28 healthy subjects to establish control ranges. The activity ranged from 1 to 7.4 nmol/min/mg protein, with a mean (±SD) value of 2.5 ± 1.7 nmol/min/mg protein. Enzyme activity was also determined in the two obligate carriers to validate the method. P39 and P40 presented residual activity levels of 24% and 32% of the control value, respectively.

The enzyme activity in lymphocytes was clearly impaired (<10% intra-assay control (IAC)) in 14 individuals with abnormal NBS results. All presented biallelic pathogenic defect variants, ranging from 0.7 to 8.2% IAC (P1–P12, P26, and P27). Patients homozygous for the most frequent mutation, c.985A > G (*n* = 9), presented the least MCAD activity (<6% IAC) (Table 1).

**Figure 1:**
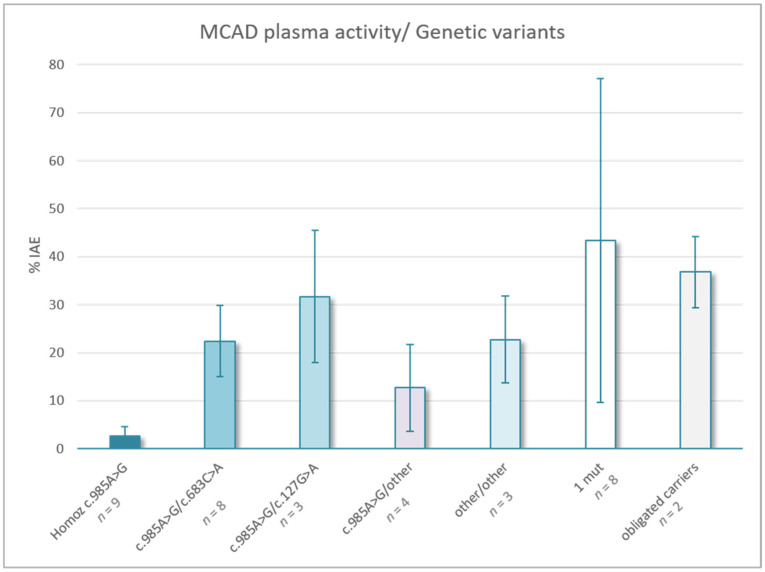
Enzymatic activity and correlation with genetic background of the patients.

Patients with combined variants c.985 A > G and c. 683C > A presented residual activity ranging from 11 to 31%. The patient bearing a LoF variant (Gln38Term) and p.Thr228Asn presented 31% activity. Patients bearing c.985G > A and c.127G > A presented residual activity ranging from 16 to 41%. The remaining patients with two pathogenic variants presented intermediate activity (13–21% IAC) (Figure 1).

Regarding cases with only one exonic variant detected, we can distinguish a group with residual activity of more than 22%, and two cases with residual activity close to 15%.

### 3.3. Clinical and Biochemical Outcomes and Treatment

Sixty-five percent of the NBS cases with two variations were asymptomatic with normal clinical parameters and growth when first referred to clinical units. Four presented minor clinical symptoms: P13, hypotonia (HP:0001252) and food intolerance (HP:0012537); P16, small gestational age (HP:0001518) with persistent creatine kinase elevation (HP:0003236) and 8% weight loss at birth (HP:0001824); P17, jaundice (HP:0000952); and P23, low birth weight (HP:0001518).

During childhood, P4 developed autistic behavior (HP:0000729), language impairment (HP:0002463), social interaction problems (HP:0000735), and macrosomia (HP:0001520). P1 at 10 years exhibits abnormal behavior (HP:0012433).

P5, P9, and P12 have been admitted to hospital several times for food intolerance (HP:0012537), vomiting (HP:0002013) or diarrhea (HP:0002014); bronchiolitis (HP:0011950); or leishmaniasis, respectively. In addition, P9, who has poor compliance with treatment, presented metabolic decompensation at 2 years of age and now presents autistic behavior (HP:0000729).

P8 was not studied by expanded NBS and attended hospital at 16 months owing to his clinical manifestations of convulsive status epilepticus (HP:0032660) and hypoglycemia (HP:0001943).

Two cases (P33 and P38) with only one variant identified presented some symptomatology. P33 presented with severe interventricular communication (HP:0010438), which was intervened, liver malfunction (HP:0001410), neurofibromatosis, coagulopathy (HP:0003256), and metabolic decompensations; P38 has had cyclical vomiting (HP:0002572) since 5 years of age, abdominal pain (HP:0002027), tiredness (HP:0012378), pallor with muscular effort (HP:0000980), and temperature episodes (HP:0001954), MCADD was suspected at 10 years of age as this case was not studied by expanded NBS.

After confirmatory studies, a dietary intervention based on avoiding prolonged fasting and medium-chain triglycerides (MCT) was recommended in those cases with two mutations or clinical symptoms. In cases with hypocarnitinemia, a maintenance dose of carnitine was supplemented, individualized according to carnitine levels [3]. The decision regarding whether or not to treat the children, and the determination of the optimal treatment for each patient, is usually based on the consensus recommendations of European experts [23].

The plasma AC levels were monitored periodically in the 30 cases with two variants and also in P33 and P38 due to their clinical manifestations. Under treatment, plasma C8 levels remained high (1–10.8 µmol/L) in cases P1–P12, P25–P27, and P30, all with residual activity (<18%). Only patients with moderate C8 levels in NBS or in their confirmatory test presented normalized levels in some of the routine AC controls.

Cases with a single variant that did not receive dietary interventions, and did not avoid long-period fasting, did not always manage to maintain C8 levels in the control range (<0.25 µmol/L).

In this cohort of patients, even though there were no major events or deaths after diagnosis by NBS, there were cases with significant adverse health outcomes.

## 4. Discussion

The detection of MCADD has been mandatory in Spanish expanded NBS programs since 2013 because early detection of this disease may improve the outcome if prophylactic measures are implemented [5], along with a significant reduction of 75% or more in metabolic decompensation disease mortality and morbidity [24,25]. However, the risk of complications remains in some patients [26,27].

Regarding our biochemical confirmatory analysis that has its own cut-off values different from NBS as samples of different nature are used (NBS uses dried blood spots and confirmation uses liquid plasma or serum), the significant differences found in the C8 concentration between homozygotes with the c.985A > G variation and compound heterozygotes with another variation, especially with c.683C > A or c.127G > A (*p* < 0.001), highlight the informative value of C8 plasma levels in a first stratification. As described in the literature, there is a correlation between the levels of C8 at diagnosis and clinical severity [28,29], and between residual activity and initial C8 levels [16].

Once genetic analysis is performed, the finding of one single pathological mutation or variations with uncertain clinical significance makes it complicated, in some cases, to achieve confirmation of the diagnosis; further, the genotype–phenotype relationship is not fully understood [30,31,32]. In our cohort, there were eight cases with only one mutation found, and taking into account the residual activity and the biochemical parameters, five of them (P31, P32, and P34–P36) could be classified as probable carriers. However, P37 and P38 presented the lowest MCAD activity among this group; in addition, P38 also presented cyclical vomiting, abdominal pain, and decay, so there is a need to discard another genetic variant not detected by the genomic approaches used. Both cases could have variants out of exonic regions asin the proximal or distal promoters, or in deep intronic regions. Alternatively, they could have other variants affecting gene expression. P33, although presenting an intermediate C8 level and MCAD activity, presented liver crisis, among other symptoms not clearly related to MCADD, so further genetic approaches are also being performed to help to elucidate the principal cause. In this group, all individuals had normal values of hexanoylglycine and suberylglycine, except for P38, who presented slight elevations on some occasions.

Concerning the phenotype/genotype correlation in homozygous patients for the loss-of-function variant c.985A > G, this group presented the most severe clinical outcomes and the lowest MCAD activity (<6% IAC). Homozygosity for the p.985A > G variant in the *ACADM* gene has been associated with a greater risk of developing clinical symptoms, but with significant phenotypic variability among homozygous patients [7]. Furthermore, these patients also showed an increase in secondary biochemical biomarkers such as acylglycines (hexanoylglycine and suberylglycine). Previous studies have shown that about 55% (range: 37–71%) of patients with MCAD deficiency prospectively identified by NBS were homozygous for the common p.985A > G pathogenic variant, consistent with our findings [7,29].

Regarding the eight cases that are compound heterozygous for c.985A > G and c.683C > A, this genetic variation is reported as a conflicting variant in ClinVar. In our patients, intermediate MCAD activity was detected, and it could be assumed that c.683C > A [p.Thr228Asn] has only a modest effect in raising plasma C8 levels, as also occurred in [33]. Most of them remain asymptomatic or mildly symptomatic, and only two presented hypocarnitinemia. This variant in combination with a null variant exhibited 31% activity. Overall, c.683C > A could be considered a milder pathogenic variant.

Concerning c.127G > A, all three patients also exhibited intermediate MCAD activity and C8 levels. Most of them remain asymptomatic, except P23, who also presented the lowest activity of the group and hypocarnitinemia. Thus, this variant could be also considered a mild one.

The remaining patients (P21–P30) are compound heterozygotes of different nucleotide variations and, as could be expected, are heterogeneous. Most of them presented significant elevations of C8 in DBS and plasma and intermediate MCAD activity, except for P25 and P26, who had increased acylglycines and also showed less than 10% IAC activity, suggesting a severe presentation of the illness. In both cases, their mutations c.250C > T and c.346T > G have not been the subject of functional studies described in the literature and are classified as VUS in ClinVar; these patients showed similar MCAD activity to homozygote patients for the most frequent mutation, highlighting the importance of knowing the residual activity to emphasize the importance of treatment and follow-up in these patients. Similarly, P21 and P22 presented the highest activity of all the patients with two biallelic mutations, which could provide important information about the prognosis of the illness.

The results presented here allowed us to stratify the true cases into two categories: (i) The first comprises 14 cases, namely, those homozygous for c.985A > G (P1–P12, P26 and P27) with high increased C8 levels at diagnosis and follow-up analysis, and severely impaired enzyme activity (<10% of IAC). These constitute the most severe presentation of the deficiency and require treatment and follow-up. (ii) The second comprises 16 patients (P13–P25 and P28–P30), forming a genetically heterogeneous group, with moderate increases in C8 levels at diagnosis and follow-up, and intermediate activity in lymphocytes (<41% IAC). This group can be classified as presenting milder forms of the disease; most of them remain asymptomatic or mildly symptomatic. Strong statistically significant differences in MCAD activity between severe cases and mild form patients (*p* < 0.001) have been calculated.

The patients’ stratification presented in this work has been based mainly on biochemical/genetic confirmation data and NBS C8 levels as we do not own C8/C2 and C8/C10 ratios of all the national neonatal screening units. This information could be very informative as it is supposed to be predictive of severe cases and could help to achieve more conclusions.

In summary, the results presented here indicate that the addition of the non-invasive enzyme activity to the diagnostic algorithm improves the diagnosis capacity, especially in cases with VUS variants or even in unsolved cases where only one variant is detected, adding information to determine whether further genetic analysis beyond exome sequencing, such as transcriptomic, methylomic, or genomic sequencing using long reads, might be performed to conclude diagnosis. In addition, analysis of the enzymatic activity can provide clues to determine clinical outcomes and to apply future tailored treatments.

## Figures and Tables

**Table 1:** Clinical, biochemical, and molecular findings in MCADD cases.

	C8 (DBS) NBS 48 h	C8 Confirmatory Test CEDEM (pl)	Nucleotide Variation	Protein Effect	Act	Act (Lf)	Therapy	Clinical Outcome
	NR: 0.16 µmol/L	mean (range) NR < 0.25 µmol/L			nmol/min/mg prot	**% IAC**		
**P1**	**8.4**	**5.73 (4–8.9)**	**c.985A > G/c.985A > G**	**p.Lys329Glu/p.Lys329Glu**	**0.06**	**5.9**	**C, AF**	**CD**
**P2**	**10.6**	**5.43 (4.1–6.7)**	**c.985A > G/c.985A > G**	**p.Lys329Glu/p.Lys329Glu**	**0.04**	**5.8**	**C, AF**	**AS**
**P3**	**15.2**	**5.85 (1.7–12.8)**	**c.985A > G/c.985A > G**	**p.Lys329Glu/p.Lys329Glu**	**0.05**	**4.5**	**C, AF**	**AS**
**P4**	**7.2**	**2.89 (0.87–5.2)**	**c.985A > G/c.985A > G**	**p.Lys329Glu/p.Lys329Glu**	**0.06**	**4.2**	**C, AF**	**AB, LR, MC**
**P5**	**11.4**	**5.9 (2–11)**	**c.985A > G/c.985A > G**	**p.Lys329Glu/p.Lys329Glu**	**0.16**	**2.6**	**C, AF**	**FI, V**
**P6^7^**	**6.6**	**9.6 (8.1–12.8)**	**c.985A > G/c.985A > G**	**p.Lys329Glu/p.Lys329Glu**	**0.09**	**1.7**	**C, AF**	**FH**
**P7^6^**	**7.2**	**3.8 (3.4–4)**	**c.985A > G/c.985A > G**	**p.Lys329Glu/p.Lys329Glu**	**0.09**	**1.7**	**C, AF**	**AS**
**P8**	**NP**	**10.88 (7.2–17.9)**	**c.985A > G/c.985A > G**	**p.Lys329Glu/p.Lys329Glu**	**0.03**	**1.2**	**C, AF**	**CSE, FH, CD (d16 m)**
**P9**	**13.7**	**7(2.4–12.5)**	**c.985A > G/c.985A > G**	**p.Lys329Glu/p.Lys329Glu**	**0.09**	**1.3**	**PC**	**AB (d2 y)**
**P10^11^**	**20**	**9.78 (9.1–10)**	**c.985A > G/c.985A > G**	**p.Lys329Glu/p.Lys329Glu**	**0.08**	**1.1**	**C, AF**	**AS**
**P11^10^**	**7.9**	**6.72 (5.7–8.2)**	**c.985A > G/c.985A > G**	**p.Lys329Glu/p.Lys329Glu**	**0.05**	**0.7**	**C, AF**	**AS**
**P12**	**8.1**	**9.9**	**c.985A > G/c.985A > G**	**p.Lys329Glu/p.Lys329Glu**	**0.05**	**0.7**	**C, AF**	**AS**
**P13^18^**	**0.39**	**0.35 (0.35–0.36)**	**c.985A > G**/c.683C > A	**p.Lys329Glu**/p.Thr228Asn	**0.34**	**30.6**	**AF**	**H, FI**
**P14**	**0.65**	**0.32 (0.17–0.6)**	**c.985A > G**/c.683C > A	**p.Lys329Glu**/p.Thr228Asn	**1.10**	**28.7**	**C, AF**	**AS**
**P15**	**0.93**	**0.45 (0.28–0.65)**	**c.985A > G**/c.683C > A	**p.Lys329Glu**/p.Thr228Asn	**0.36**	**28.8**	**C, AF**	**AS**
**P16**	**1.19**	**0.33 (0.2–0.4)**	**c.985A > G**/c.683C > A	**p.Lys329Glu**/p.Thr228Asn	**0.62**	**26.7**	**AF**	**LGW, CK**
**P17^20^**	**0.37**	**0.25 (0.11–0.33)**	**c.985A > G**/c.683C > A	**p.Lys329Glu**/p.Thr228Asn	**0.24**	**21.6**	**AF**	**J**
**P18^13^**	**1.56**	**0.35 (0.21–0.46)**	**c.985A > G**/c.683C > A	**p.Lys329Glu**/p.Thr228Asn	**0.24**	**16.9**	**AF**	**AS**
**P19**	**0.67**	**0.49 (0.34–0.64)**	**c.985A > G**/c.683C > A	**p.Lys329Glu**/p.Thr228Asn	**0.54**	**15**	**AF**	**AS**
**P20^17^**	**NP**	**0.31**	**c.985A > G**/c.683C > A	**p.Lys329Glu**/p.Thr228Asn	**0.38**	**11**	**AF**	**AS**
**P21**	**0.61**	**0.57 (0.32–0.82)**	**c.985A > G**/c.127G > A	**p.Lys329Glu**/p.Glu43Lys	**0.45**	**40.5**	**AF**	**AS**
**P22**	**0.38**	**0.23 (0.16–0.3)**	**c.985A > G**/c.127G > A	**p.Lys329Glu**/p.Glu43Lys	**1.50**	**38.8**	**AF**	**AS**
**P23**	**0.47**	**0.43 (0.33–0.52)**	**c.985A > G**/c.127G > A	**p.Lys329Glu**/p.Glu43Lys	**0.16**	**15.8**	**C, AF**	**LWB, AS**
**P24^35^**	**1.24**	**0.46**	**c.985A > G**/c.599+3A > G	**p.Lys329Glu**/p.?	**0.59**	**22**	**AF**	**AS**
**P25**	**3.89**	**2 (1.3–2.7)**	**c.985A > G**/G626C > T	**p.Lys329Glu**/p.Pro209Leu	**0.18**	**18.3**	**C, AF**	**AS**
**P26**	**3.43**	**5.17 (2.5–8.9)**	**c.985A > G**/c.250C > T	**p.Lys329Glu**/p.Leu84Phe	**0.46**	**8.2**	**C, AF**	**AS**
**P27**	**6.0**	**1.5**	**c.985A > G**/c.346T > G	**p.Lys329Glu**/p.Cys116Gly	**0.03**	**2.4**	**C, AF**	**AS**
**P28**	**1.03**	**0.3 (0.21–0.46)**	c.683C > A/c.999_1011 dup13	p.Thr228Asn/p.Gln338Term	**0.56**	**31**	**C, AF**	**AS**
**P29**	**0.48**	**0.2 (0.08–0.48)**	c.351A > C/c.503A > C	p.Thr117Thr/p.Asp168Ala	**0.45**	**24**	**AF**	**AS**
**P30**	**2.31**	**1.0 (0.34–1.5)**	c.338C > A/c.940G > C	p.Ala113Asp/p.Val314Leu	**0.53**	**13.1**	**C, AF**	**AS**
**P31**	**0.5**	**0.12**	**c.985A > G**	**p.Lys329Glu**	**1.42**	**100**	**No**	**AS**
**P32**	**0.5**	**0.79**	c.253G > C	p.Gly85Arg	**1.10**	**9.5**	**No**	**FH**
**P33**	**0.49**	**0.34 (0.08–0.73)**	c.449_452delCTGA	p.Thr150Argfs*4	**1.27**	**37**	**AF**	**LC, Ds**
**P34**	**0.17**	**0.57**	**c.985A > G**	**p.Lys329Glu**	**0.81**	**34**	**No**	**AS**
**P35^24^**	**0.12**	**0.27**	**c.985A > G**	**p.Lys329Glu**	**0.79**	**30**	**No**	**AS**
**P36**	**0.43**	**0.22**	**c.985A > G**	**p.Lys329Glu**	**0.51**	**22.3**	**No**	**AS**
**P37**	**0.42**	**0.11**	**c.985A > G**	**p.Lys329Glu**	**0.28**	**16**	**No**	**AS**
**P38**	**NP**	**0.27 (0.11–0.53)**	**c.985A > G**	**p.Lys329Glu**	**0.48**	**14.7**	**AF**	**CV, AP, D, P, TE (d10 y)**
**P39^m3^**	**NP**	**NP**	**c.985A > G**	**p.Lys329Glu**	**1.72**	**42**	**No**	**/**
**P40^f35^**	**NP**	**NP**	**c.985A > G**	**p.Lys329Glu**	**0.73**	**32**	**No**	**/**

NR: normal range, NP: not performed, IAC: intra-assay control, Lf: lymphocytes, pl: plasma sample >1 month, DBS: dot blood spots. Therapy: C: carnitine suppl, AF: Avoid fasting PC: Poor compliance. clinical outcome: AB: autistic behavior, LR: languaje retardation, MC: macrosomy, CD: conduct disorder, LGW: low gestational weight, CK: creatine kinase, AS: asymptomatic, J: jaundice, H: Hypotonia, FI: food intolerance, LWB: low weight at birth, LC: liver crisis, FH: fasting hypoglucemias, V: vomiting, CV: cyclical vomiting, AP: abdominal pain, D: decay, P: pallor, TE: temperatur episodes, CSE: convulsive status epilepticus. Ds: decompensaion 2 years.
